# Elevated asprosin in hypertension: evidence from an exploratory case-control study

**DOI:** 10.1038/s41598-025-32824-y

**Published:** 2026-01-03

**Authors:** Hana Alkhalidy, Aseel Assamak, Islam Al-Shami, Tareq L. Mukattash, Tuqa Khashman, Dongmin Liu

**Affiliations:** 1 Department of Human Nutrition, Foods and Exercise, College of Agriculture and Life Sciences, Virginia Tech, Blacksburg, VA 24061 USA; 2 Department of Nutrition and Food Technology, Faculty of Agriculture, Jordan University of Science and Technology, P.O. Box 3030, Irbid, 22110 Jordan; 3 Department of Clinical Nutrition and Dietetics, Faculty of Applied Medical Science, The Hashemite University, Zarqa, Jordan; 4 Department of Clinical Pharmacy, Faculty of Pharmacy, Jordan University of Science and Technology, Irbid, 22110 Jordan

**Keywords:** Hypertension, Asprosin, Biomarker, Diagnosis, Biomarkers, Cardiology, Diseases, Endocrinology, Medical research, Risk factors

## Abstract

Hypertension (HTN) is a major risk factor of cardiovascular diseases (CVDs). Asprosin, a novel adipokine, has been linked to metabolic diseases; however, its role in HTN remains unclear. This study investigated the association between asprosin levels and HTN in 110 sex-matched participants. Sociodemographic, lifestyle, and family history data were collected, and anthropometric measurements and fasting blood samples were obtained to assess glucose, asprosin, and insulin levels. Insulin resistance and β-cell function were estimated using LogHOMA-IR and LogHOMA-β. Statistical analyses included t-tests, chi-square, Mann-Whitney U tests, receiver operating characteristic analysis with area under the curve (AUC), and ridge-penalized logistic regression. Cases reported higher stress levels and a greater maternal history of HTN compared with controls. Blood glucose, insulin, LogHOMA-IR and LogHOMA-β were all significantly elevated in cases. Median asprosin levels were higher in cases (98.7 ng/ml, IQR 57.7) than controls (65.4 ng/ml, IQR 55.4; *p* < 0.001) and demonstrated good discriminatory ability for HTN (AUC = 0.827; cut-off point > 79.15 ng/ml), with notable sex differences, as males showed higher discriminatory ability compared with females. Elevated asprosin was associated with higher odds of HTN (β = 1.31, SE = 0.319; OR = 3.71, 95% CI: 1.99–6.93), though the association attenuated after adjusting for antihypertensive medication and other confounders. These findings suggest that asprosin may serve as a potential biomarker for HTN, warranting further investigation into its clinical relevance.

**Supplementary Information:**

The online version contains supplementary material available at 10.1038/s41598-025-32824-y.

## Introduction

Hypertension (HTN) is a chronic disease characterized by persistently elevated systolic and diastolic blood pressure (SBP and DBP) affecting approximately 1.4 billion adults worldwide^1^. Between the years 1990 and 2019, the prevalence of HTN increased by 41% among European and American adults, and by 144% among South-East Asia and Western Pacific region adults^2^. Among Jordanians adults, the prevalence increased by 22%^3^. Despite its growing prevalence, the exact causes of HTN remain unclear. However, it is mainly found that it stems from abnormalities in several biological systems. In the vascular system, factors such as blood vessels restructuring, elevated vasoreactivity and vascular dysfunction play key roles^4^. In the renal system, disruptions in the renin-angiotensin-aldosterone system and the pressure diuresis are contributing mechanisms^5^. The central nervous system may affect blood pressure through sympathetic overstimulation and signals from kidney, adipose tissue, and heart^5^. Other contributing factors include increased inflammation, oxidation from reactive oxygen species, and high dietary salt intake^5^. Furthermore, most metabolic syndromes characterized by insulin resistance (IR), abdominal obesity, and dyslipidemia are associated with HTN and cardiovascular disease (CVD) risk through pathways involving oxidative stress and inflammation^6^.

HTN is typically diagnosed using validated oscillometric upper arm cuff device as recommended by the American Heart Association^7^. Research interest has shifted toward identifying novel biomarkers that may aid in early diagnosis or improve understanding of HTN pathophysiology^8^. For instance, Cystatin C, a protein biomarker, has shown promise in predicting pre-capillary pulmonary HTN^9^, yet its clinical utility in essential HTN is limited. Cystatin C is associated to renal function^10^ and demonstrated sex-specific association; being linked to HTN in women but not in men without chronic kidney disease^11^. Another biomarker, the chemokine CXCL13, has been associated with essential HTN through elevated gene expression, and may contribute to disease pathogenesis^12^. Nonetheless, CXCL13 may lack specificity due to its broad involvement in inflammatory process^13^. Asprosin, a glucogenic hormone, has gained attention as a potential metabolic mediator due to its association with metabolic syndrome and chronic diseases^14^. Elevated asprosin levels have been positively correlated with coronary artery disease (CAD)^15–17^ potentially through mechanisms mediated by IR, excessive adiposity and hyperlipidemia^16^. With respect to HTN, a pre-clinical study in rats suggested that asprosin influence vascular inflammation and remodeling^18^. Another pre-clinical study found that asprosin affected sympathetic activity and blood pressure regulation through paraventricular nervous system^19^. However, despite its links to metabolic abnormalities, the relationship between asprosin levels and HTN in humans has not yet been established. Therefore, the present study aims to investigate the association between asprosin levels and HTN in a Jordanian adult population. Furthermore, it seeks to determine the sociodemographic and lifestyle factors, and anthropometric measures that may influence this association. Understanding these associations may contribute to the development of novel approaches for HTN diagnosis, prevention and treatment.

## Methods

### Study design

This case-control study was conducted between October 2022 and January 2023 with subjects recruited at King Abdullah University Hospital in northern Jordan. Participants were selected based on predefined inclusion and exclusion criteria.

Control group: Adults (aged ≥ 18 years), non-pregnant and non-lactating females, who reported no prior diagnosis of HTN or other chronic diseases (including cancer, thyroid diseases, and endocrinological disorder) by a healthcare provider. Controls were also not taking medications for HTN, thyroid disorders, diabetes, dyslipidemia or hormonal therapies (including oral contraceptive).

Case group: Adults with self-reported, physician-diagnosed HTN^20^ and who indicated the current use of antihypertensive medication. To minimize confounding from metabolic or chronic illness, cases reported no prior diagnosis of diabetes, liver diseases, renal diseases, or cancer by a healthcare provider.

Cases and controls were matched by sex with a ratio of 1:1. The study was conducted in accordance with the ethical guidelines of the Declaration of Helsinki and received approval from the Institutional Review Board at Jordan University of Science and Technology (2022/152/25). Written informed consent was obtained from all participants.

### Data collection

Subjective data were collected via face-to-face interviews using a structured questionnaire administered by a trained researcher. The questionnaire included sections on sociodemographic characteristics, medical history, selected lifestyle factors, and parental medical history. Physical activity was assessed using the International Physical Activity Questionnaire (IPAQ) in Arabic language^21^. The levels of physical activities were calculated, and physical activity was classified into low, moderate or high. Stress was measured using Perceived Stress Scale with 14 items (PSS-14) Questionnaire in Arabic language and the answers were classified into low, moderate or high stress levels^22^.

For objective data, body mass index (BMI) was calculated in (kg/m^2^)^23^ after obtaining weight and height as described below and classified according to World Health Organization guidelines^24^. Weight was measured using a calibrated digital scale while subjects wore light clothes^25^ and recorded to the nearest 0.1 kg. Height was measured using stadiometer, with participants barefoot, standing straight with heels together, and head in the Frankfort horizontal plane^25^ and recorded to the nearest 0.5 cm.

Waist circumference (WC) was measured using a non-elastic and flexible tape at the narrowest point between the lowest ribs and superior iliac crest. It was recorded to the nearest 0.1 cm^26^. Enlarged WC was defined as larger than 102 cm for males and 88 cm for females^27^.

Fasting blood glucose (FBG) was measured using a glucometer after overnight fast for at least 8 h. FBG was classified according to American Diabetic Association (ADA) criteria: less than 100 mg/dL (normal), 100–125 mg/dL (prediabetes), and equal or more than 126 mg/dL (diabetes)^28^.

Blood pressure was measured using a sphygmomanometer with participants seated and resting for 5 min. Two readings were taken at heart level in mmHg and averaged for analysis^29^.

### Serum hormones measurements

Blood samples were collected using serum-separating tubes. After clotting, samples were centrifuged to obtain serum then stored at -20 C until analysis. Asprosin levels were measured ELISA kit (Abcam, U.S.) according to manufacture protocol with a sensitivity of 0.92 ng/ml^30^. Fasting insulin (FINS) was measured using an ELISA assay kit (Mercodia, Sweden) with a sensitivity of ≤ 1 mU/L^31^. Homeostatic model assessment of IR (HOMA-IR) and β-cell function (HOMA-β) were calculated as described by Matthews et al.^32^. However, they were represented and analyzed as log transformed to reduce their effects of skewness^33^.

### Statistical analysis

Sample size was calculated using G*power, assuming a 1:1 case to control ratio. Because no prior studies had reported comparable effect sizes for asprosin in relation to HTN, we used a medium effect size (Cohen’s f^2^ = 0.15) based on Cohen’s conventions^34^. With a power of 80%^35^ and a type 1 error rate of α = 0.05, and a confidence interval (CI) of 95%, the required sample size was determined to be 55 participants per group.

Continuous variables were tested for normality distribution using Shapiro-Wilk test, and by examining skewness and kurtosis^36^. Normally distributed data are presented as mean (standard deviation (SD)) whereas non-normally distributed data are expressed as median (interquartile range (IQR)). Categorical variables are summarized as numbers and frequencies. Differences between groups were assessed using chi-square tests for categorical variables, the independent t-test for normally distributed continuous variables and Mann-Whitney U test for non-normally distributed.

Correlation analyses were conducted to examine the relationship between asprosin and anthropometric measures (WC and BMI). For asprosin analyzed as a continuous variable, we applied a rank-based partial Spearman correlation to account for non-normally distributions and adjust for case-control status^37^. This approach involved ranking each continuous variable, regressing the ranked variable on case-control group, and computing the Spearman correlation between the resulting residuals^38^. Values of ρ ≈ 0.10, 0.30, and 0.50 are considered small, medium, and large effects, respectively in which Spearman’s ρ follows the same interpretation as Pearson’s r as both are standardized measures ranging from − 1 to 1^39,40^. In addition, we used a partial point biserial correlation (partial Pearson) adjusted for case–control status between the binary variable asprosin cut-off points and continuous anthropometric measures (BMI and WC)^41^ and values of *r* < 0.30 were considered as weak, r 0.30–0.49 as moderate, and *r* ≥ 0.50 as strong^41^.

The receiver operating characteristic (ROC) curve was used to determine the cut-off values for asprosin in relation to HTN.

Ridge-penalized logistic regression was used to examine the association between asprosin levels (low vs. high) and HTN risk, with HTN status as the dependent variable and adjusting for the predictors. This approach was chosen instead of logistic regression due to the relatively small sample size, unstable maximum likelihood estimates^42^, and the presence of multicollinearity among predictors that was tested through variance inflation factor (see Supplementary Table 2) as a multicollinearity diagnostic tool with values of < 5 and tolerance < 0.2 as threshold for acceptable collinearity^43^. Ridge regression stabilizes model estimation by shrinking coefficients toward zero, thereby reducing variance and improving overall model robustness^44,45^. A penalty parameter of λ = 1 was applied, which is commonly used for small to moderate sample sizes to enhance model stability^45^.

The ridge-penalized regression model was fitted with different models of predictors^46^ as follows: model 1: unadjusted model; model 2: adjusting for sociodemographic (age, sex, educational level, marital status, and family income); model 3: adjusted for anthropometric variables (BMI, BMI categories, WC, WC categories, and BMI and WC risk categories); model 4: adjusted for biochemical parameters (FINS, FBG, LogHOMA-IR, LogHOMA-β, and FBG categories); model 5: adjusted for lifestyle factors (physical activity level, stress level, and smoking status); model 6: adjusted for parental history of CVDs and HTN; model 7: adjusted for HTN medications; model 8: adjusted for all previous adjusted models (2–7).

Results were reported as penalized odds ratios and penalized confidence intervals were calculated from the penalized information without reporting p values because penalized regression do not provide valid standard errors or hypothesis tests^47^.

No missing data was presented. All analyses were performed using IBM statistical package for social science (SPSS) software for windows, Version 21.0. (IBM Corp., Armonk, NY, U.S.) and validated Python-based syntax implemented within the SPSS software was used for penalized logistic regression analysis.

## Results

**Fig. 1:**
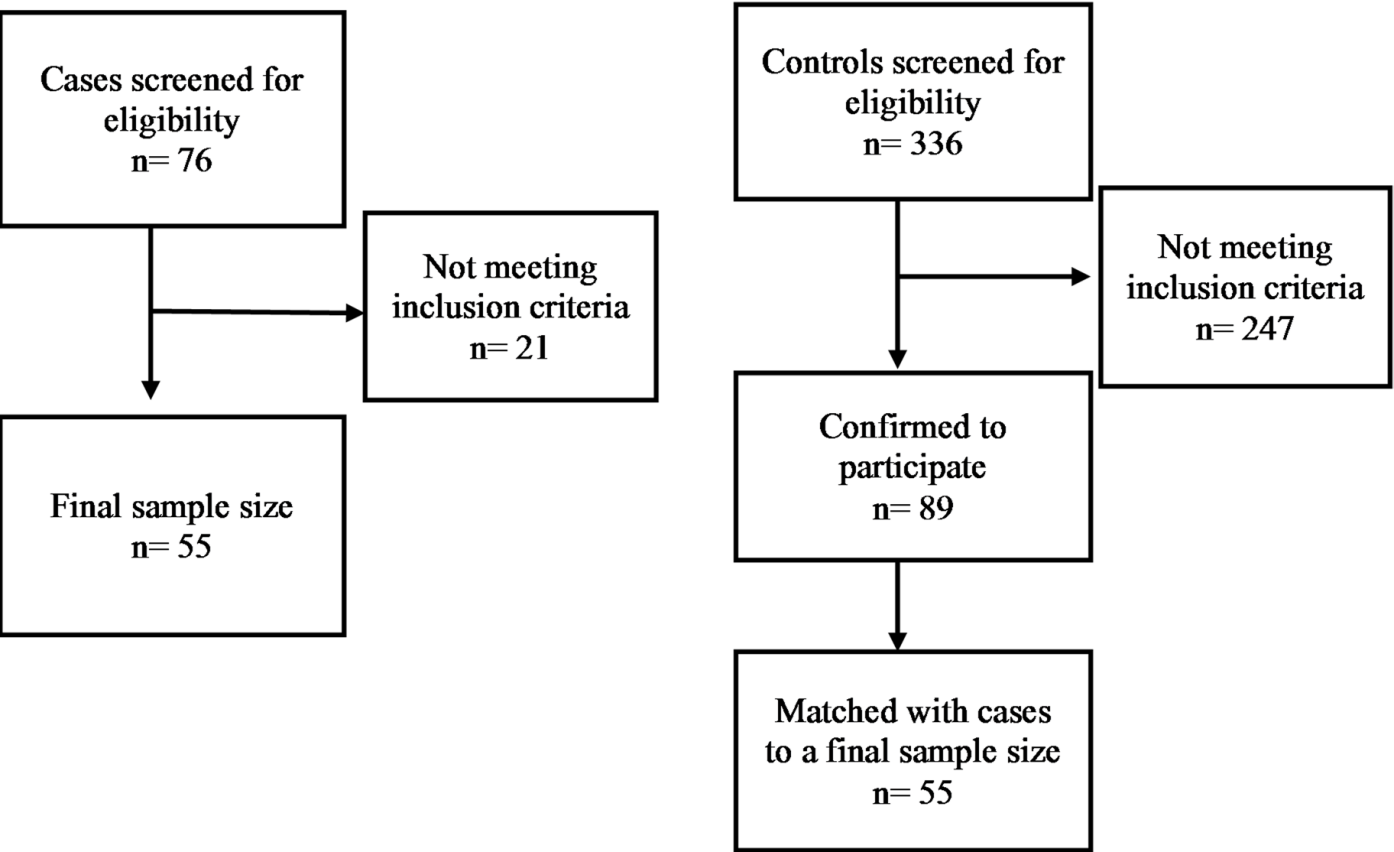
sample size and recruitment for cases and controls.

The study included 110 participants, divided equally into two groups: cases: 55 individuals with HTN and controls: 55 healthy individuals without any known chronic medical conditions (Fig. 1). The mean measured SBP and DBP values were higher in among cases (SBP: 131.4 ± 15.5 and DSP: 87.2 ± 15.8 mmHg) as expected being closer to elevated blood pressure values^29^. These measurements were not significantly different when compared to the control group (SBP: 125.4 ± 16.0 and DBP: 82.5 ± 10.4 mmHg) (*p* = 0.053 for SBP, *p* = 0.067 for DBP) among total population. When stratified by sex, male participants with HTN showed a significantly higher SBP (131.41 ± 14.8 mmHg) compared with male controls (123.9 ± 12.0 mmHg; *p* = 0.047). While DBP did not differ significantly between the two groups (cases: 87.7 ± 11.1 mmHg and controls: 83.8 ± 7.8; *p* = 0.15). Among female participants, neither SBP (cases:131.33 ± 16.8 mmHg and controls: 126.7 ± 18.9 mmHg) nor DBP (cases: 86.7 ± 20.4 mmHg and controls: 81.3 ± 12.2 mmHg) showed significant differences between cases and controls.

The sociodemographic characteristics were generally comparable between groups (Table 1). The mean age was similar between groups (49.16 ± 6.8 for controls vs. 49.92 ± 9.2 for cases, *p* > 0.05)). No significant differences were observed in marital status, educational level, or family income between the two groups.

**Table 1:** Sociodemographic characteristics for the study’s population.

Variable	Controls			Cases			*P* value
	Total	Males	Females	Total	Males	Females	
Age (years)	49.16 (6.8)	48.52 (15.0)¥	50.98 (5.4)	49.92 (9.2)	50.50 (8.1)	49.17 (10.6)	0.623
Marital status n (%)							0.952
Single	4 (7.3)	3 (11.5)	1 (3.4)	4 (7.3)	3 (9.7)	1 (4.2)	
Married	47 (85.5)	23 (88.5)	24 (82.8)	46 (83.6)	27 (87.1)	19 (79.2)	
Divorced/Separated	4 (7.2)	0 (0.0)	4 (13.8)	5 (9.1)	1 (3.2)	4 (16.6)	
Educational level n (%)							0.089
High school or less	20 ( 36.3)	8 (30.8)	12 (41.4)	29 (52.7)	12 (38.7)*	17 (70.8)*	
Bachelor or Diploma	26 (47.3)	11 (42.3)	15 (51.7)	23 (41.8)	16 (51.6)*	7 (29.2)*	
Postgraduate	9 (16.4)	7 (26.9)	2 (6.9)	3 (5.5)	3 (9.7)*	0 (0.0)*	
Family income n (%)							0.275
< 350 JOD	8 (14.6)	2 (7.7)	6 (20.7)	16 (29.1)	5 (16.1)	11 (45.9)	
350–800 JOD	34 (61.8)	17 (65.4)	17 (58.6)	28 (50.9)	20 (64.5)	8 (33.3)	
> 800 JOD	13 (23.6)	7 (26.9)	6 (20.7)	11 (20.0)	6 (19.4)	5 (20.8)	

Age was expressed as ¥ median (IQR) or mean (SD) and categorical data were expressed as number of participants n (% within the one group).

Independent sample t-test was used for comparison between normally distributed continuous variables, independent sample Mann-Whitney U test for non-normally distributed continuous variables and Chi square test was used for categorical variables.

JOD: Jordanian Dinar.

*p < 0.05 Within group (males vs. females within case, males vs. females within control).

#*p* < 0.05 Between groups (same sex: case vs. control).

Table 2 presents the participants’ anthropometric measurements including BMI, WC, and their respective risk categories. In addition, combinations of BMI with WC are presented to assess chronic disease risk. No significant differences were found between cases and controls for any of these measures. The study population exhibited elevated anthropometric measures with BMI values of 30.7 kg/m^2^ (IQR: 9.5) for controls and 33.2 kg/m^2^ (IQR: 6.8) for cases, (*p* > 0.05) and WC of 101.2 ± 12.4 cm for controls and 105.9 ± 14.6 cm for cases (*p* > 0.05). However, when stratified by sex, male participants with HTN had a significantly higher BMI compared to male controls (*p* < 0.05).

**Table 2:** Anthropometric measures for the study’s population.

Variable	Controls			Cases			*P* value
	Total	Males	Females	Total	Males	Females	
BMI	30.7 (9.5)¥	27.7 (5.8)¥#**	34.1 (6.1)**	33.2 (6.8)	**32.0 (7.4)#**	34.6 (5.6)	0.160
WC	101.2 (12.4)	98.5 (16.5)	101.0 (12.4)	105.9 (14.6)	106.7 (14.9)	104.9 (14.4)	0.074
BMI categories n (%)							0.274
Under weight	0 (0.0)	0 (0.0)*	0(0.0)*	1 (1.8)	1 (3.2)	0 (0.0)	
Normal weight	5 (9.1)	4 (15.4)*	1 (3.4)*	4 (7.3)	3 (9.7)	1 (4.2)	
Overweight	22 (40.0)	14 (53.8)*	8 (27.6)*	14 (25.5)	9 (29.0)	5 (20.8)	
Obese	28 (50.9)	8 (30.8)*	20 (69.0)*	36 (65.4)	18 (58.1)	18 (75.0)	
WC categories n (%)							0.145
Normal	20 (36.4)	15 (57.7)**	5 (17.2)**	13 (23.6)	10 (32.3)	3 (12.5)	
Enlarged	35 (63.6)	11 (42.3)**	24 (82.8)**	42 (76.4)	21 (67.7)	21 (87.5)	
Elevated BMI&WC n (%)							0.145
No risk	20 (36.4)	15 (57.7)**	5 (17.2)**	13 (23.6)	10 (32.3)	3 (12.5)	
At risk	35 (63.6)	11 (42.3)**	24 (82.8)**	42 (76.4)	21 (67.7)	21 (87.5)	

Continuous variables were expressed as ¥ median (IQR) or mean (SD) and categorical data were expressed as number of participants n (% within the one group).

Independent sample t-test was used for comparison between normally distributed continuous variables, independent sample Mann-Whitney U test for non-normally distributed continuous variables, and Chi square test was used for categorical variables.

Underweight: subjects’ BMI < 18.5 kg/m^2^, normal weight: subjects’ BMI 18.5–24.9 kg/m^2^, overweight: subjects’ BMI 25.0–29.9 kg/m^2^ and obese: subjects’ BMI > 29.9 kg/m^2^.

Enlarged WC: males’ WC > 102 cm and females’ WC > 88 cm.

BMI: Body Mass Index; WC: Waist Circumference.

*p < 0.05 Within group (male vs. female within case, male vs. female within control).

**p < 0.01Within group (male vs. female within case, male vs. female within control).

#*p* < 0.05 Between groups (same sex: case vs. control).

Table 3 demonstrates the participants’ biochemical measurements. Asprosin levels were significantly higher in the case group (98.7 ng/ml (IQR: 57.7)) compared to controls (65.4 ng/ml (IQR: 55.4)), (*p* < 0.001). Similarly, FINS levels were significantly higher in cases (10.8 µIU/mL (IQR: 9.1)) compared to controls (7.3 µIU/mL (IQR: 5.2), (*p* < 0.01). FBG was also significantly higher among cases (5.4 ± 0.66 mmol/L) versus controls (5.3 mmol/L (IQR: 0.61)), (*p* < 0.05). Additionally, LogHOMA-IR and LogHOMA-β values were significantly higher in cases (0.87 ± 0.67 and 4.7 ± 0.76, respectively) compared to controls (0.52 ± 0.51 and 4.4 ± 0.48, respectively; both *p* < 0.05). Based on the ADA criteria, 72.7% of controls had normal measured FBG while 27.3% had values of pre-diabetic range, with no diabetic cases. In contrast, only 40.0% of cases had normal FBG; 41.8% were in the prediabetic range, and 18.2% met criteria for diabetes (*p* < 0.001). Significant differences were observed between cases and controls within the same sex (Table 3).

**Table 3:** Biochemical measurements for the study’s population.

Variable	Controls			Cases			*P* value
	Total	Males	Females	Total	Males	Females	
Asprosin ng/ml	65.4 (55.4)¥	**73.3 (30.3)###**	**66.4 (68.4)¥###**	98.7 (57.7)¥	**125.5 (71.0)¥###**	**112.0 (37.2)###**	**0.00**
FINS µIU/mL	7.3 (5.2)¥	**7.5 (3.8)#**	**6.9 (5.2)¥**	10.8 (9.1)¥	**13.4 (8.7)#**	8.9 (3.5)	**0.018**
FBG mmol/L	5.3 (0.61)¥	5.3 (0.44)	**5.3 (0.56)¥#**	5.4 (0.66)	5.4 (0.74)	**6.1 (1.6)#**	**0.001**
LogHOMA-IR^a^	0.52 (0.51)	**0.45 (0.54)#**	0.58 (0.49)	0.87 (0.67)	**0.95 (0.72)#**	0.66 (0.46)	**0.021**
LogHOMA-β^a^	4.4 (0.48)	**4.3 (0.51)#**	4.42 (0.45)	4.7 (0.76)	**4.8 (0.85)#**	4.5 (0.49)	**0.044**
Asprosin level categories n (%)							**0.000**
Normal	36 (65.5)	18 (69.2) **###**	18 (62.1)##	7 (12.7)	**3 (9.7)###**	**4 (16.7)##**	
High	19 (34.5)	8 (30.8) **###**	11 (37.9)##	48 (87.3)	**28 (90.3)###**	**20 (83.3)##**	
FBG categories n (%)							**0.000**
Normal	40 (72.7)	**18 (69.2)#**	**22 (75.9)##**	22 (40.0)	**13 (41.9)#**	**9 (37.5)##**	
Prediabetes	15 (27.3)	**8 (30.8)#**	**7 (24.1)##**	23 (41.8)	**12 (38.7)#**	**11 (45.8)##**	
Diabetes	0 (0.0)	**0 (0.0)#**	**0 (0.0)##**	10 (18.2)	**6 (19.4)#**	**4 (16.7)##**	

Continuous variables were expressed as ¥ median (IQR) or mean (SD) and categorical data were expressed as **number** of participants n (% within the one group).

Independent sample t-test was used for comparison between normally distributed continuous variables, independent sample Mann-Whitney U test for non-normally distributed continuous variables, and Chi Square test was used for categorical variables. FINS: Fasting insulin; FBG: Fasting blood glucose; HOMA-IR: Homeostasis Model Assessment of Insulin Resistance; HOMA-β: Homeostasis Model Assessment of pancreatic beta cells. *: *p* < 0.05 Within group (male vs. female within case, male vs. **female** within control). #: *p* < 0.05 Between groups (same sex: case vs. control). ##: *p* < 0.01 Between groups (same sex: case vs. control). ###: *p* < 0.001 Between groups (same sex: case vs. control). ^**a**^Natural-log–transformed variables.

Lifestyle factors and parental medical history among the study population are summarized in Table 4. Physical activity levels did not differ significantly between the two groups and most participants reported moderate to high activity levels. However, perceived stress levels showed a significant difference. Among controls, 27.3% reported low stress, 67.2% moderate stress, and 5.5% high stress. Whereas among cases, 18.2% reported low stress, 56.4% moderate stress, and 25.4% high stress. Males in the case group reported significantly higher stress levels compared with males in control group (*p* < 0.05). Smoking status did not differ between the groups, with 29.1% of controls and 27.3% of cases identifying as current smokers, and 3.6% of controls and 10.9% of cases reporting past smoking. Similarly, no significant differences were observed between cases and controls regarding the family history of CVDs. However, maternal history of HTN was reported more frequently among cases, especially among females, compared with controls (*p* < 0.05).

**Table 4:** Lifestyle characteristics and parental history of CVD and HTN for the study’s population.

Variable	Controls			Cases			*P* value
	Total	Males	Females	Total	Males	Females	
Physical activity level n (%)							0.269
Low	10 (18.2)	6 (23.1)	4 (13.8)	17 (30.9)	14 (45.2)***	3 (12.5)***	
Moderate	26 (47.3)	14 (53.8)	12 (41.4)	20 (36.4)	14 (45.2)***	6 (25.0)***	
High	19 (34.5)	6 (23.1)	13 (44.8)	18 (32.7)	3 (9.6)***	15(62.5)***	
Stress level n (%)							**0.013**
Low	15 (27.3)	**10 (38.5)#**	5 (17.3)	10 (18.2)	**5 (16.1)#**	5 (20.8)	
Moderate	37 (67.2)	**16 (61.5)#**	21 (72.4)	31 (56.4)	**20 (64.5)#**	11 (45.8)	
High	3 (5.5)	**0 (0.0)#**	3 (10.3)	14 (25.4)	**6 (19.4)#**	8 (33.4)	
Smoking status n (%)							0.34
Non-smoker	37 (67.3)	11 (42.3)**	26 (89.7)**	34 (61.8)	11 (35.5)***	23 (95.8)***	
Smoker	16 (29.1)	13 (50.0)**	3 (10.3)**	15 (27.3)	15 (48.4)***	0 (0.0)***	
Past-smoker	2 (3.6)	2 (7.7)**	0 (0.0)**	6 (10.9)	5 (16.1)***	1 (4.2)***	
Fathers have HTN n (%)	13 (23.6)	6 (23.1)	7 (24.1)	19 (34.5)	11 (35.5)	8 (33.3)	0.208
Fathers have CVDs n (%)	14 (25.5)	4 (15.4)	10 (34.5)	17 (30.9)	10 (32.3)	7 (29.2)	0.525
Mothers have HTN n (%)	23 (41.8)	9 (34.6)	**14 (48.3)##**	36 (65.5)	16 (51.6)*	20 (83.3)##*	**0.013**
Mothers have CVDs n (%)	14 (25.5)	2 (7.7)**	12 (41.4)**	14 (25.5)	7 (22.6)	7 (29.2)	1.00

Categorical data expressed as number of participants n (% within the one group).

Chi square test was used for categorical variables.

* :*p* < 0.05 Within group (male vs. female within case, male vs. female within control).

**: *p* < 0.01 Within group (male vs. female within case, male vs. female within control).

***: *p* < 001 Within group (male vs. female within case, male vs. female within control).

#: *p* < 0.05 Between groups (same sex case vs. same sex control).

##: *p* < 0.01 Between groups (same sex case vs. same sex control).

HTN: hypertension; CVDs: cardiovascular diseases.

The ROC curve analysis evaluating the predictive performance of asprosin levels for HTN is presented in Table 5 and Supplementary Figs. 1–3. In the overall population, the ROC analysis demonstrated good predictive performance of asprosin for HTN (AUC = 0.827, with cut-off point of > 79.15 ng/ml, sensitivity of 87.27, and specificity of 65.45), (*p* < 0.001). Sex-stratified analyses showed notable differences with males demonstrating a higher discriminatory ability (AUC = 0.862, with cut-off point of > 77.07 ng/ml, sensitivity of 90.32, and specificity of 69.23), (*p* < 0.001) compared with females (AUC = 0.810, with cut-off point of > 67.10 ng/ml, sensitivity of 95.83,  and specificity of 55.17), (*p* < 0.001).

**Table 5:** Area under ROC curve (AUC), optimal cut-off values, sensitivities, and specificities of Asprosin levels in predicting HTN.

Variables	AUC (95% CIs)	Cut-off	Sensitivity (95% CIs)	Specificity (95%CIs)	Youden index J	*p*-value
Total population	0.827 (0.743–0.892)	> 79.15	87.27	65.45	0.527	< 0.001
Males	0.862 (0.745–0.939)	> 77.07	90.32	69.23	0.596	< 0.001
Females	0.810 (0.678–0.904)	> 67.10	95.83	55.17	0.51	< 0.001

Optimal cut-off values were determined using the Youden index. AUC values are shown with (95% CI) and statistical significance was set at *p* < 0.05. AUC: area under the curve; CI: confidence interval.

Correlation analysis was conducted between asprosin and both BMI and WC prior to regression, given that obesity measures may influence this adipokine. However, no significant associations were observed between asprosin and BMI or between asprosin and WC (Supplementary Table 1).

Ridge-penalized logistic regression was performed using several adjustment models (Table 6). In the unadjusted model (Model 1), higher Asprosin levels were associated with increased odds of HTN (β = 1.31, SE = 0.319; OR = 3.71, 95% CI: 1.99–6.93). After adjusting for the sociodemographic variables (age, sex, family income, educational level, and marital status) in Model 2, the association strengthened (β = 1.58, SE = 0.384; OR = 4.85, 95% CI: 2.28–10.3). Additional analyses adjusting sex and age individually indicated that neither variable independently affected the association. Adjustment for anthropometric variables (BMI, BMI categories, WC, WC categories, BMI and WC risk categories) in Model 3 produced stable estimates (β = 1.28, SE = 0.331; OR = 3.60, 95% CI: 1.88–6.88). In contrast, adjusting for biochemical parameters (FINS, FBG, LogHOMA-IR, LogHOMA-β, and FBG categories) in Model 4 slightly reduced the association (β = 1.22, SE = 0.423; OR = 3.39, 95% CI: 1.48–7.78). Model 5, which adjusted for lifestyle factors (physical activity level, stress level, and smoking status) yielded higher estimates for the association between elevated asprosin and HTN (β = 1.66, SE = 0.441; OR = 5.25, 95% CI: 2.22–12.5) with stress and smoking identified as contributing factors to the increase in OR. Adjustment for parental history of CVDs and HTN in Model 6 resulted in stabilized estimates (β = 1.31, SE = 0.336; OR = 3.69, 95% CI: 1.91–7.13). Interestingly, when use of antihypertensive medications was added in Model 7, the association weakened (β = 0.737, SE = 0.5; OR = 2.09, 95% CI: 0.785–5.56). In the final fully adjusted model (Model 8), which included all variables from Models 2–7, the estimates slightly declined (β = 0.623, SE = 0.791; OR = 1.87, 95% CI: 0.396–8.78).

**Table 6:** Associations of Asprosin levels with HTN among the study population.

Model	Coef β	SE	OR	95% CI
1	1.31	0.319	3.71	1.99–6.93
2	1.58	0.384	4.85	2.28–10.30
3	1.28	0.331	3.60	1.88–6.88
4	1.22	0.423	3.39	1.48–7.78
5	1.66	0.441	5.25	2.22–12.5
6	1.31	0.336	3.69	1.91–7.13
7	0.737	0.500	2.09	0.785–5.56
8	0.623	0.791	1.87	0.396–8.78

Data is derived from ridge-penalized logistic regression, the ordinary hypothesis tests (p-values) and standard confidence intervals are not appropriate. Coefficients (β) and odds ratios (OR = e^β) are descriptive indicating the direction and relative strength of associations.

Model 1: unadjusted model; model 2: adjusting for sociodemographic (age, sex, educational level, marital status, and family income); model 3: adjusted for anthropometric variables (BMI, BMI categories, WC, WC categories, and BMI and WC risk categories); model 4: adjusted for biochemical parameters (FINS, FBG, LogHOMA-IR, LogHOMA-β, and FBG categories; model 5: adjusted for lifestyle factors (physical activity level, stress level, and smoking status); model 6: adjusted for parental history of CVDs and HTN; model 7: adjusted for HTN medications; model 8: adjusted for all previous adjusted models (2–7).

β: Penalized coefficient; SE: standard error; OR: odd ratio; CI: confidence interval.

## Discussion

Our findings showed that asprosin levels were significantly higher among individuals with HTN compared to healthy controls. This association was observed despite the absence of differences in sociodemographic characteristics between groups. Anthropometric variables including BMI and WC and their respective risk categories, were also comparable, indicating that the observed elevation in asprosin was not attributable to differences in obesity status. In contrast, the metabolic parameters; FBG, FINS, LogHOMA-IR, and LogHOMA-β levels, were all significantly higher in individuals with HTN, supporting previous evidence linking asprosin to metabolic abnormalities^48^. Elevated asprosin has been positively correlated with HOMA-IR, hemoglobin A1c (HbA1c), FBG and FINS in individuals with type 2 diabetes^49–51^ and with triglycerides, albumin, FBG and HOMA-IR in individuals with non-alcoholic fatty liver disease^52^. Among lifestyle factors, perceived stress showed a significant difference, being higher among cases. Additionally, a greater proportion of individuals with HTN reported maternal history of HTN suggesting a possible familial or maternal contribution to HTN risk^53^.

Penalized ridge regression was employed due to the small sample size^44,45^, and to avoid unstable and inflated ORs produced by ordinary logistic regression. The regression analyses demonstrated that individuals with high asprosin levels (> 79.15 ng/ml) had higher ORs of HTN, even after adjusting for a wide range of confounding variables including sociodemographic, anthropometric, biochemical, lifestyle, and familial variables. The inclusion of antihypertensive medication use attenuated this association, suggesting that medication may influence asprosin levels or modify pathways linking asprosin to blood pressure regulation. Antihypertensive drugs have been shown to influence adipokine profiles^54,55^ and such effects may partially explain this attenuation. ROC analysis further showed that asprosin had a good discriminatory ability for HTN in the overall population (AUC = 0.827), with a cut-off value of 79.15 yielding high sensitivity (87.3%) and moderate specificity (65.5%). However, its performance should be interpreted with caution given the influence of medication.

Sex is a crucial biological factor affecting blood pressure regulation, primarily due to the modulatory effects of sex hormones on the renin–angiotensin–aldosterone system, which differ significantly between males and females^56^. To account for this, we matched participants by sex ensuring more accurate and reliable findings and also stratified analyses by sex. These sex-specific analyses demonstrated some notable differences. Among males with HTN, SBP and BMI were higher compared with male controls, whereas such differences were not observed among females. Additionally, maternal history of HTN was more prevalent among female cases compared with female controls. Asprosin exhibited higher discriminatory ability for predicting HTN in males (AUC = 0.862) than in females (AUC = 0.810) suggesting sex-related differences in its regulation or relevance. Variations in hormonal profiles, fat mass and distribution, and their related metabolic responses^57^ may underlie these findings and warrant further investigation^58^.

Preclinical studies provide mechanistic hypothesis linking asprosin to HTN pathophysiology^18,19^. In animal models, hypertensive rats had increased asprosin expression in the aorta and mesenteric arteries. When asprosin was knocked down, NOD-like receptor thermal protein domain associated protein 3 inflammasome expression was attenuated. It also, resulted in structural improvements in vascular tissues, including increased lumen diameter and decreased medial thickness in the carotid artery^18^. Asprosin was highly expressed in the paraventricular nucleus of the brain and may influence sympathetic nervous system activity and blood pressure regulation. Microinjection of asprosin in this brain region increased sympathetic outflow and blood pressure through production of reactive oxygen species (ROS) and NAPDH oxidase activation via cAMP-protein kinase A signaling pathway^19^. These pathways suggest possible biological links between asprosin and HTN; however, translating these mechanisms from animal models to human physiology is challenging and require caution, as several factors must be addressed to be ensure reliability and validity^59^.

Associations between asprosin and broader CVDs have been reported^16,60,61^. For instance, In a study, elevated asprosin levels were independently associated with heart failure severity, contributing to vascular remodeling and enlargement of cardiac chambers (OR: 1.010, 95% CI: 1.003–1.018, *p* < 0.05)^61^. In another study, asprosin was proposed as a biomarker for CAD (AUC = 0.870, cut-off point = 6.05 µg/ml, sensitivity = 78.4, specificity = 76.1; *p* < 0.001). Adjusting for confounders including age, sex and BMI resulted in an OR of 3.01 for predicting CAD (95% CI: 2.16–4.20, *p* < 0.001)^16^. Similarly, significant difference in asprosin levels were reported between individuals with acute coronary syndrome compared to controls (5.27 ± 0.67 ng/ml vs. 3.82 ± 1.2 ng/ml, *p* < 0.001), with strong diagnostic performance (AUC; 0.95, sensitivity; 94%, specificity; 85%) ^60^. Given the shared pathophysiological mechanisms underlying these conditions and HTN^62^, the present findings aligns with the emerging literature suggesting a role for asprosin in cardiometabolic abnormalities. However, considering the design of the present study and the broader literature, the directionality of the association remains uncertain. Elevated asprosin may reflect a causal mechanism, a compensatory physiological response, or a secondary marker of underlying shared metabolic dysfunction.

This study has several strengths including a sufficiently powered sample, with participants matched by sex and comprehensive adjustment for clinical, biochemical, lifestyle, and familial variables. Several limitations should also be considered. The sample was recruited from a single medical center, which may limit generalizability. Although individuals with known physician-diagnosed diabetes were excluded based on self-report, FBG measurements collected during the study revealed that a proportion of participants in the HTN group met the criteria for diabetes. These cases likely represent undiagnosed diabetes, a common occurrence in individuals with HTN^63,64^, but they may still introduce metabolic heterogeneity. To mitigate this, regression models adjusted for glycemic markers, yet the possibility of residual confounding cannot be fully excluded. Additionally, SBP and DBP values were higher in the case group, the differences did not reach statistical significance, likely due to the use of antihypertensive medications, which can lower blood pressure measurements^65^ and reduce between-group differences despite an underlying diagnosis of HTN. Medication use may also attenuate the association between asprosin and HTN, as observed in the adjusted regression models, highlighting the need for careful consideration of treatment effects in biomarker research. Additionally, although the sample size was adequate, the smaller sex-stratified subgroups may have limited the stability of sex-specific estimates, particularly in the ROC analyses. Finally, because cases were defined based on physician-diagnosed HTN rather than untreated or newly diagnosed elevated BP, measured BP at the time of data collection may not fully reflect disease status.

In conclusion, our study demonstrates that asprosin levels are significantly elevated in individuals with HTN, independent of sociodemographic and anthropometric factors, and are associated with metabolic abnormalities. Asprosin exhibited good discriminatory ability for HTN and remained positively associated with HTN after adjusting for a wide range of confounders, though antihypertensive medication use attenuated this association. These findings support the potential of asprosin as a novel biomarker for HTN and highlight the importance of considering sex differences and medication effects in clinical interpretations. Future studies with larger, multi-center cohorts, including newly diagnosed HTN and longitudinal follow-up are needed to clarify the independent role of asprosin in HTN and its potential relevance as a biomarker.

## Supplementary Information

Below is the link to the electronic supplementary material.

Supplementary Material 1

Supplementary Material 2

## Data Availability

All data generated or analyzed during this study are included in this published article and its Supplementary information files.
