# Supplementary material for: Elevated asprosin in hypertension: evidence from an exploratory case-control study
Source: Sci Rep. 2026 Jan 3;16:2973. doi: 10.1038/s41598-025-32824-y (PMC12830809; doi:10.1038/s41598-025-32824-y)
Supplement: Supplementary file 1 — Supplementary Material 1 [file 41598_2025_32824_MOESM1_ESM.docx]

STROPE checklist for “Elevated asprosin in hypertension: evidence from an exploratory case-control study” manuscript

STROBE Statement—checklist of items that should be included in reports of observational studies

|  | Item No. | Recommendation | Page  No. | Relevant text from manuscript |
| --- | --- | --- | --- | --- |
| **Title and abstract** | 1 | (*a*) Indicate the study’s design with a commonly used term in the title or the abstract | 1 | Title section, line 1 |
|  |  | (*b*) Provide in the abstract an informative and balanced summary of what was done and what was found | 1 | Abstract section, lines 2-16 |
| Introduction | | | |  |
| Background/rationale | 2 | Explain the scientific background and rationale for the investigation being reported | 2-3 | Introduction section, lines 22-60. |
| Objectives | 3 | State specific objectives, including any prespecified hypotheses | 3 | Introduction section, lines 55-60. |
| Methods | | | |  |
| Study design | 4 | Present key elements of study design early in the paper | 3 | Methods section, lines 63. |
| Setting | 5 | Describe the setting, locations, and relevant dates, including periods of recruitment, exposure, follow-up, and data collection | 3-5 | Methods section, lines 63-104 |
| Participants | 6 | (*a*) *Cohort study*—Give the eligibility criteria, and the sources and methods of selection of participants. Describe methods of follow-up  *Case-control study*—Give the eligibility criteria, and the sources and methods of case ascertainment and control selection. Give the rationale for the choice of cases and controls  *Cross-sectional study*—Give the eligibility criteria, and the sources and methods of selection of participants | 3-4 | Methods section, lines 62-75. |
|  |  | (*b*) *Cohort study*—For matched studies, give matching criteria and number of exposed and unexposed  *Case-control study*—For matched studies, give matching criteria and the number of controls per case | 4-5 | Methods section, page 4, line 69 and page 5, lines 103-105 |
| Variables | 7 | Clearly define all outcomes, exposures, predictors, potential confounders, and effect modifiers. Give diagnostic criteria, if applicable | 4-6 | Methods section, lines 76-121 |
| Data sources/ measurement | 8* | For each variable of interest, give sources of data and details of methods of assessment (measurement). Describe comparability of assessment methods if there is more than one group | 4-5 | Methods section, lines 76-104 |
| Bias | 9 | Describe any efforts to address potential sources of bias | 6 | Methods section, lines 118-119 |
| Study size | 10 | Explain how the study size was arrived at | 5 | Methods section, lines 106-108 |

| Quantitative variables | 11 | Explain how quantitative variables were handled in the analyses. If applicable, describe which groupings were chosen and why | 4-5 | Methods section, lines 79-104 |
| --- | --- | --- | --- | --- |
| Statistical methods | 12 | (*a*) Describe all statistical methods, including those used to control for confounding | 5-6 | Methods section, lines 105-121 |
|  |  | (*b*) Describe any methods used to examine subgroups and interactions | 6 | Methods section, lines 118-119 |
|  |  | (*c*) Explain how missing data were addressed | 6 | Methods section, lines 119-120 |
|  |  | (*d*) *Cohort study*—If applicable, explain how loss to follow-up was addressed  *Case-control study*—If applicable, explain how matching of cases and controls was addressed  *Cross-sectional study*—If applicable, describe analytical methods taking account of sampling strategy | 4 | Methods section, line 72 |
|  |  | (*e*) Describe any sensitivity analyses | - | - |
| Results | | | | |
| Participants | 13* | (a) Report numbers of individuals at each stage of study—eg numbers potentially eligible, examined for eligibility, confirmed eligible, included in the study, completing follow-up, and analysed | 6 | Results section, lines 123-124 |
|  |  | (b) Give reasons for non-participation at each stage | 20 | Figure section, lines 414-416 |
|  |  | (c) Consider use of a flow diagram | 20 | Figures section, lines 414-416 |
| Descriptive data | 14* | (a) Give characteristics of study participants (eg demographic, clinical, social) and information on exposures and potential confounders | 6-8 | Results section, lines 124-156 |
|  |  | (b) Indicate number of participants with missing data for each variable of interest | - | - |
|  |  | (c) *Cohort study*—Summarise follow-up time (eg, average and total amount) | - | - |
| Outcome data | 15* | *Cohort study*—Report numbers of outcome events or summary measures over time |  |  |
|  |  | *Case-control study—*Report numbers in each exposure category, or summary measures of exposure | 7 | Results section, lines 138-139 |
|  |  | *Cross-sectional study—*Report numbers of outcome events or summary measures |  |  |
| Main results | 16 | (*a*) Give unadjusted estimates and, if applicable, confounder-adjusted estimates and their precision (eg, 95% confidence interval). Make clear which confounders were adjusted for and why they were included | 8 | Results section, lines 163-171 |
|  |  | (*b*) Report category boundaries when continuous variables were categorized | 8 | Results section, lines 157-162 |
|  |  | (*c*) If relevant, consider translating estimates of relative risk into absolute risk for a meaningful time period | - | - |

| Other analyses | 17 | Report other analyses done—eg analyses of subgroups and interactions, and sensitivity analyses |  | Supplementary tables file |
| --- | --- | --- | --- | --- |
| Discussion | | | | |
| Key results | 18 | Summarise key results with reference to study objectives | 8-9 | Discussion section, lines 173-186 |
| Limitations | 19 | Discuss limitations of the study, taking into account sources of potential bias or imprecision. Discuss both direction and magnitude of any potential bias | 10 | Discussion section, lines 218-220 |
| Interpretation | 20 | Give a cautious overall interpretation of results considering objectives, limitations, multiplicity of analyses, results from similar studies, and other relevant evidence | 9-10 | Discussion section, lines 187-215 |
| Generalisability | 21 | Discuss the generalisability (external validity) of the study results | 10 | Discussion section, lines 219-220 |
| Other information | |  | | |
| Funding | 22 | Give the source of funding and the role of the funders for the present study and, if applicable, for the original study on which the present article is based | - | - |

*Give information separately for cases and controls in case-control studies and, if applicable, for exposed and unexposed groups in cohort and cross-sectional studies.

**Note:** An Explanation and Elaboration article discusses each checklist item and gives methodological background and published examples of transparent reporting. The STROBE checklist is best used in conjunction with this article (freely available on the Web sites of PLoS Medicine at http://www.plosmedicine.org/, Annals of Internal Medicine at http://www.annals.org/, and Epidemiology at http://www.epidem.com/). Information on the STROBE Initiative is available at www.strobe-statement.org.
