# Supplementary material for: Elevated asprosin in hypertension: evidence from an exploratory case-control study
Source: Sci Rep. 2026 Jan 3;16:2973. doi: 10.1038/s41598-025-32824-y (PMC12830809; doi:10.1038/s41598-025-32824-y)
Supplement: Supplementary file 2 — Supplementary Material 2 [file 41598_2025_32824_MOESM2_ESM.docx]

**
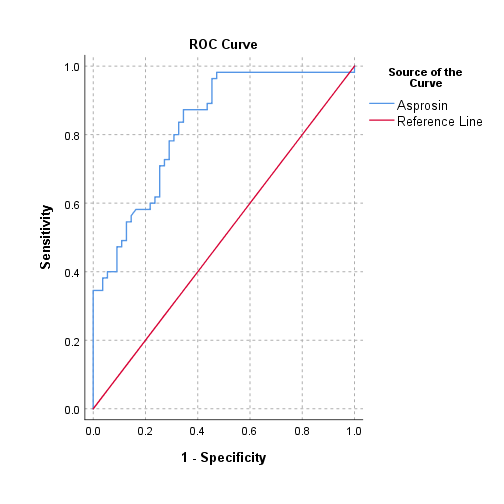
**

**Supplementary Figure 1: ROC curve for asprosin cut-off points among total population**

**
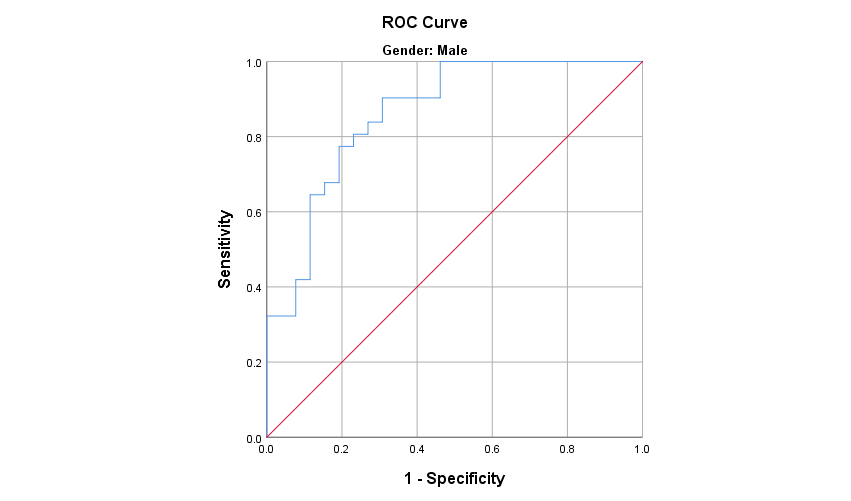
**

**Supplementary Figure 2: ROC curve for asprosin cut-off points among males**

**
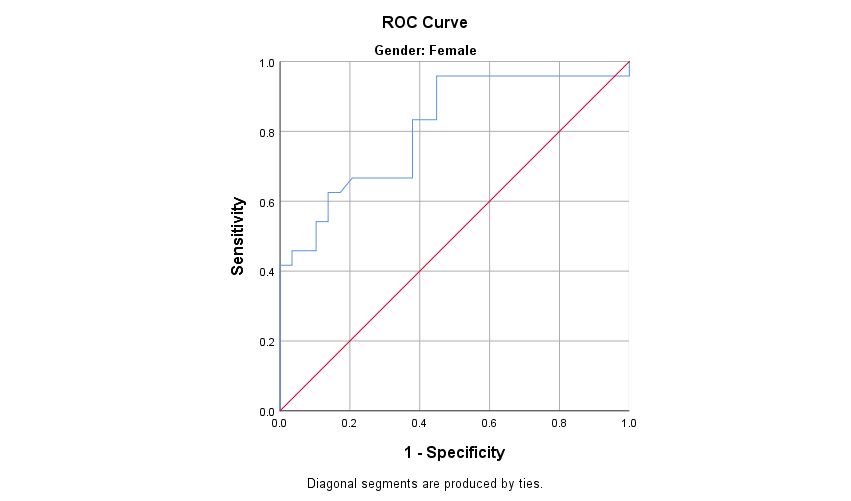
**

**Supplementary Figure 3: ROC curve for asprosin cut-off points among females**

**Supplementary Table 1: correlation analysis of asprosin with anthropometric measures**

|  | **BMI** | **WC** |
| --- | --- | --- |
| **Asprosin level*** | 0.064 (p=0.50) | 0.01 (p=0.992) |
| **Asprosin cut-off points#** | -0.026 (p=0.79) | -0.048 (p=0.62) |
| *Rank-based residual (regressed) partial Spearman correlation; ρ ≈ 0.10, 0.30, and 0.50 indicate small, medium, and large effects, respectively that Spearman’s ρ follows the same interpretation as Pearson’s r because both are standardized measures ranging from −1 to 1 (1, 2)  #Partial point-biserial correlation; values r< 0.30 were considered as weak, r 0.30-0.49 as moderate, and r ≥ 0.50 as strong (3)  Asprosin levels, BMI and WC were continuous, while asprosin cut-off points was categorical of high: value of >79.15 ng/ml and low: values ≤79.15  BMI: body mass index; WC: waist circumference | | |

**Supplementary Table 2: Variance inflation factor for multicollinearity diagnosis**

| Predictor | Collinearity Statistics | |
| --- | --- | --- |
|  | Tolerance | VIF |
| Asprosin cut off points | 0.55 | 1.81 |
| Age | 0.69 | 1.45 |
| Sex | 0.39 | 2.55 |
| Marital status | 0.60 | 1.67 |
| Educational level | 0.53 | 1.87 |
| Family’s income | 0.53 | 1.90 |
| BMI (Kg/m^2^) | 0.20 | 5.11 |
| BMI categories | 0.24 | 4.23 |
| WC (cm) | 0.21 | 4.73 |
| BMI and WC risk categories | 0.35 | 2.86 |
| FINS | 0.11 | 9.44 |
| LogHOMA_IR | 0.11 | 9.15 |
| logHOMA_β | 0.17 | 6.00 |
| FBG (mmol/L) | 0.58 | 1.73 |
| FBG categories | 0.42 | 2.41 |
| Physical activity level | 0.63 | 1.58 |
| Stress level | 0.60 | 1.67 |
| Smoking status | 0.52 | 1.94 |
| Fathers have HTN | 0.70 | 1.43 |
| Fathers have CVDs | 0.78 | 1.29 |
| Mothers have HTN | 0.69 | 1.45 |
| Mothers have CVD | 0.76 | 1.31 |
| HTN medications | 0.49 | 2.05 |
| Multicollinearity was assessed using VIF. Values of VIF< 5 and tolerance< 0.2 were considered acceptable for none major collinearity (4)  VIF: variance inflation factor; BMI: body mass index; WC: waist circumference; FINS: fasting insulin; LogHOMA-IR: Log-transformed Homeostatic Model Assessment of Insulin Resistance; logHOMA_β: Log-transformed Homeostatic Model Assessment of β-cell Function; FBG: fasting blood glucose; HTN: hypertension; CVDs: cardiovascular diseases | | |

References:

1. Cohen, J. A power primer. *Psychol. Bull*. **112**, 155–159 (1992).

2. Hauke, J. & Kossowski, T. Comparison of values of Pearson's and Spearman's correlation coefficients on the same sets of data. *Quaest. Geogr.* **30**, 87–93 (2011).

3. Hinkle, D. E., Wiersma, W. & Jurs, S. G. *Applied statistics for the behavioral sciences* (Houghton Mifflin Boston, 2003).

4. O’Brien, R. M. A caution regarding rules of thumb for variance inflation factors. *Qual. Quant.* **41**, 673–690 (2007).
